# Demographic and Socioeconomic Determinants of Body Mass Index in People of Working Age

**DOI:** 10.3390/ijerph17218168

**Published:** 2020-11-05

**Authors:** Daniel Puciato, Michał Rozpara

**Affiliations:** 1 Faculty of Physical Education and Physiotherapy, Opole University of Technology, ul. Prószkowska 76, 45-758 Opole, Poland; 2 Institute of Sport Sciences, The Jerzy Kukuczka Academy of Physical Education, ul. Mikołowska 72A, 40-065 Katowice, Poland; m.rozpara@awf.katowice.pl

**Keywords:** hedging, transaction costs, dynamic programming, risk management, postdecision state variable

## Abstract

Obesity is currently the most common metabolic disease, causing numerous health problems and, if untreated, leading to premature mortality. Obesity is a significant issue among people of working age since their ability to work depends directly on their health condition and psychomotor fitness. Demographic and socioeconomic factors have a significant impact on the body weight of people of working age. The aim of this study is to identify relationships between the body mass index and selected demographic and socioeconomic variables in working-age residents of the city of Wrocław, Poland. The study involved 4315 respondents (2206 women and 2109 men) aged 18–64 years from Wrocław. The sample selection was random and purposive, using multilevel stratification. The applied research tool was the authors’ own cross-sectional diagnostic questionnaire of socioeconomic status. Based on the collected data, the respondents’ body weight was categorized according to WHO criteria. The majority of respondents (60%) had a normal body weight, while 40% were categorized as overweight or obese. The difference was statistically significant (*p* < 0.001). Sex, age, occupational status, marital status, number of people in the household, having a steady source of income, disposable (net) income, and savings were significantly correlated (*p* < 0.001) with respondents’ body mass index. Public health programs aimed at promoting healthy lifestyle behaviors should be addressed primarily to groups at the highest risk of overweight and obesity.

## 1. Introduction

Obesity is currently the most prevalent metabolic disease and has been formally recognized as a global epidemic of the 21st century. According to World Health Organization data [1], 38.9% of the global population over 18 years old are overweight, of which 13.1% are obese. The problem of overweight is particularly visible in highly developed countries, for example, in North America, where it amounts to 67.5%. In Europe, the percentage of overweight people is about 58.7%, and in Poland, 60.0% of adults are overweight and 25.0% are obese [2].

Excess body mass has been a well-documented cause of premature death. Study results indicate that the all-cause mortality rate is at least 20% higher among obese adults than among normal-weight adults [3]. Excess body mass causes many metabolic and hormonal disorders, and a particularly strong correlation exists between obesity and type 2 diabetes [4]. In obese individuals, major disorders of the circulatory system can also be observed, including cardiac adiposity, arteriosclerotic lesions in arterial and coronary vessels, blood hypervolemia and hyperviscosity, and excessive heart workload [5]. Within the respiratory system, obesity is often associated with decreased lung volume and obstructive sleep apnea [6]. It also results in a number of adverse changes in the musculoskeletal system as it involves decreased muscle strength and endurance and the necessity to undertake long-term muscle work [7]. Consequently, obesity leads to degenerative changes in the osteoarticular system and increased lower back pain [8]. Researchers also revealed the negative effects of obesity on the function of the reproductive system [9] and on the skin [10]. Increased intracellular metabolic processes, associated with excess body fat accumulation, may also lead to a number of oncological conditions [11]. Relationships of excessive body mass with mental and social health status have also been proven. Obesity has been shown to be often associated with anxiety, depression symptoms, aggressive behaviors, or low self-esteem [12]. It is also accompanied by deteriorated social functioning and decreased health-related quality of life [13].

Overweight is a particularly important problem for people of working age as their work ability depends directly on their health condition and psychomotor fitness [14]. Obesity may indirectly contribute to the occurrence of negative social and economic consequences on both a microscale (employees, households, business companies) and a macroscale (national economies). The main effects of obesity on employees themselves may encompass more frequent and longer absenteeism from work, limited professional performance, or lower incomes. Similar strains on businesses include a decline in labor productivity, an increasing need to contribute to employees’ sickness benefits, or problems with work organization. The negative consequences of obesity in people of working age for the national economy can be increased healthcare costs, a reduced number of professionally active people, lower state tax revenues, and, consequently, a lower economic growth rate [15]. The global economic burden of obesity is very high, and it roughly amounts to $2.0 trillion [15]. This represents approximately 2.8% of global GDP [16].

Earlier population studies have indicated that body weight is affected by factors such as age, gender, place of residence, level of education, marital status, family situation, professional status, or financial situation [17,18,19,20,21,22,23,24]. Several studies [25,26,27,28,29] to date have used aggregated indices of socioeconomic status and estimated the directions and strength of the impact of these indices on body weight measured with the body mass index (BMI). Pigeyre et al. [25] and Christine et al. [26] revealed negative correlations between BMI and socioeconomic status, while Wagner et al. [27] found these correlations only among women. On the other hand, Akinyemiju et al. [28] and Araujo et al. [29] noted positive correlations between BMI and socioeconomic status.

Therefore, despite some earlier observations, the relationship between BMI and socioeconomic status is still not fully explained, and socioeconomic status determinants, such as having a steady income, disposable (net) income, savings, and debt, have not been considered. So far, the research on BMI and socioeconomic status has only focused on selected age groups: children and adolescents, adults, or the elderly. However, the relationship between the BMI and socioeconomic status in people of working age, in the broad age bracket of 18–64 years [30,31], has been rarely investigated. Meanwhile, people in this age range constitute the majority of labor resources, which are of key importance for the functioning of societies and economies.

The present study attempts to close the indicated research gaps by considering a scope of demographic and socioeconomic variables as determinants of body weight, assessed with the BMI in people of working age.

The aim of the study is to identify the relationships between the body mass index and selected demographic and socioeconomic variables in working-age residents of the city of Wrocław, Poland. Two research questions have been formulated:

1. What percentage of Wrocław working-age respondents are characterized by normal body weight, according to WHO criteria?
2. Are age, gender, education, occupational status, marital status, number of people in the household, having a steady source of income, gross and net income, debt, and savings associated with the respondents’ BMI?

## 2. Materials and Methods

### 2.1. Participants

The study was conducted in Wrocław, Poland. The research sample comprised 4315 people (2206 women and 2109 men) aged 18–64, i.e., about 1% of the working-age residents of Wrocław.

The sample selection was random and purposive, using a three-level stratification. First, with the use of a random number table, ten residential areas were selected from all alphabetically ordered Wrocław neighborhoods. Secondly, three streets from each selected residential area were chosen using a random number table. Thirdly, every fourth person, selected from among passers-by in the chosen streets, was asked to participate in the survey. The following criteria were adopted for the inclusion of participants in the study: address of residence (one of the selected streets); working age, i.e., 18–64 years; and lack of chronic diseases such as cancer, diabetes, hypertension, arthritis, or osteoporosis. All respondents were informed about the aim and the procedure of the study and asked to give their consent to filling in the study questionnaire. The research project was approved by the Commission of Bioethics of the University School of Physical Education in Wrocaw.

Table 1 presents the demographic and socioeconomic characteristics of Wrocław residents.

### 2.2. Study Design

The study was cross-sectional and conducted using a diagnostic survey questionnaire based on a direct interview technique. The interviews were conducted by the authors and their collaborators, who, if needed, explained in detail the individual questionnaire items and answers to the respondents. The main research instrument was the authors’ own questionnaire of socioeconomic status, consisting of eleven questions grouped into statements regarding demographic and socioeconomic variables. The questionnaire was prepared on the basis of the analysis of a number of studies, in particular, Akinyemiju et al. [28], Araujo et al. [29], Basto-Abreu et al. [30], Christine et al. [26], Compernolle et al. [17], Hong et al. [20], Kolahi et al. [21], Kriaucioniene et al. [23], Mbada et al. [31], Mehboob et al. [22], Micklesfield et al. [19], Pigeyre et al. [25], Puciato et al. [15], Rengma et al. [24], Stewart-Knox et al. [18], and Wagner et al. [27].

Prior to the main part of the study, the reliability of the questionnaire was assessed using the test–retest method [32]. For this purpose, a randomly selected group of 115 people (51 men, 64 women) filled in the questionnaire two times, 14 days apart. Based on the obtained results, the reliability coefficient (r = 0.987) was calculated, which confirmed the questionnaire’s reliability. The respondents’ suggestions were also used to correct the perceived errors and uncertainties in the questionnaire.

The answers to the socioeconomic status questionnaire yielded information on the respondents’ demographic variables: sex (woman, man), age (18–24, 25–34, 35–44, 45–54, 55–64), and education (primary, secondary, university). The questionnaire was also used to collect data on the respondents’ socioeconomic status, i.e., occupation (manual worker, white-collar worker, self-employed, student, unemployed), marital status (single, married), number of persons per household (1, 2, 3, 4, 5, or more), having a steady income (No, Yes), per capita income (up to 130, 131–260, 261–390, 391–520 and above 520 USD), disposable (net) income (no net income, up to 52, 53–104, 105–156 and above 156 USD), having savings (No, Yes), and debt (No, Yes).

During the interviews, data on the respondents’ height and body weight were collected. On that basis, the body mass index (BMI) was calculated for each respondent, which was then used to categorize all study participants following WHO criteria [33] into two groups: normal weight (BMI = 18.5–24.9 kg/m^2^) and overweight (BMI ≥ 25.0 kg/m^2^).

### 2.3. Statistical Analysis

The data were analyzed in terms of number and frequency of variable categories, arithmetic means, standard deviation, as well as minimum and maximum values. The differences between the selected categories of variables were checked with the chi-squared test. To assess the relationships between the body mass index and demographic and socioeconomic variables, the chi-squared test, Cramér’s V, and the binary logistic regression analysis were used. The variables fitting the model were selected using stepwise regression. The level of statistical significance was set at α = 0.05. All statistical calculations were made using IBM SPSS Statistics 26.0 (IBM Corporation, Armonk, NY, USA).

### 2.4. Ethical Approval

The research project has been approved by the Commission of Bioethics of the University School of Physical Education in Wrocław (no. 11/2016).

## 3. Results

The Wrocław residents’ mean age was 40.45 ± 13.71 years. The mean body height was 172.34 ± 10.04 cm, body weight—72.97 ± 13.92 kg, and the BMI—24.45 ± 3.45 kg/m^2^. In the whole study group, the lowest BMI was 18.5 kg/m^2^, and the highest BMI amounted to 39.0 kg/m^2^.

Table 2 presents the division of respondents according to their BMI, following WHO criteria. The majority (60%) of the respondents were of normal body weight, while 40% were overweight (*p* < 0.001).

Almost all demographic and socioeconomic variables, apart from having money savings, revealed a statistically significant correlation (*p* < 0.001) with the Wrocław residents’ body weight, as evaluated according to the WHO criteria. The values of Cramér’s V = 0.06–0.30 indicate, at the same time, the low strength of these correlations. The women were significantly more likely to be of normal body weight (71.40%) than men (48.13%). Therefore, according to WHO standards, the body weight of the majority of examined men (51.87%) was excessive. The highest percentage of individuals with normal body weight was found among respondents aged 18–24 (81.60%) and 25–34 (74.26%) years, and the lowest among respondents aged 45–54 (39.10%) and 55–64 (49.75%) years. The majority of respondents from the two oldest age groups were, therefore, characterized by excessive body weight. Considering the level of education, normal body weight was most often found in people with secondary education (64.94%) and primary education (58.45%), while it was least frequently observed in people with university education (53.97%). The highest percentage of people with normal body weight was noted among students (85.40%), and the lowest among the self-employed (48.25%). Most representatives of the latter (51.75%) were therefore characterized as overweight. Normal body weight was also noted in 60.60% of manual workers, 59.31% of the unemployed, and 54.07% of white-collar workers. The BMI values, as recommended by WHO, were also observed in 72.78% of single respondents and in 51.42% of married ones. The highest percentage of respondents with normal body weight was noted in those living in the most numerous households, i.e., with four (65.58%) and five and more persons (62.79%), and the lowest (53.51%) in two-person households. Normal body weight was observed in almost 75% of respondents without a steady income source and 57% of those with a regular income. The analysis of monthly gross income per capita showed that the highest percentage of people with normal body weight was in the two groups of respondents with the lowest income (71.53%—up to 130 USD, and 65.79%—from 131 to 260 USD), while the lowest was among those with the highest income (50.63%—above 520 USD). Similar patterns were observed in terms of disposable (net) income. The highest percentage of residents with normal body weight (72.18%) was found in those without net income, and the lowest (48.39%) in residents with the highest net income, i.e., the majority (51.61%) of the latter were overweight. BMI within the WHO normal range was noted in 61.39% of respondents having money savings and in 58.52% having no money savings. However, differences between the percentages of respondents from both groups were not statistically significant (*p* = 0.055). Additionally, 62.74% of Wrocław residents without debt and 57.01% of those in debt were found to be of normal body weight (Table 3).

Table 4 presents a logistic regression model illustrating the relationship of body mass according to WHO criteria (dependent variable) with demographic and socioeconomic characteristics (independent variables) among Wrocław residents of working age. The likelihood ratio (LR = 884.01, *p* < 0.001) and the coefficient of determination (R2_Nagelkerke_ = 0.25 for a model with eight independent variables) show that the model differs significantly from the one containing only the intercept. Sex, age, occupation, marital status, number of people in the household, having a steady source of income, net income, and savings are variables significantly correlated with normal body weight among the Wrocław residents under study. The odds ratio of normal body weight according to WHO was over 3.5 times higher in women than in men. The odds for normal body weight were 3.2 times higher in respondents aged 18–25 years, 2.4 times higher in the age group of 25–35 years, 35% higher in 36–45-year-olds, and 42% lower in respondents aged 46–55 years than in the oldest group. In comparison to the reference group, i.e., the unemployed from Wrocław, higher odds for normal body weight were found in manual workers (OR = 2.48), students (OR = 2.29), self-employed (OR = 1.78), and white-collar workers (OR = 1.62). The odds for normal body weight in the unmarried respondents were about 30% higher than in married respondents. Moreover, they were 34% higher among Wrocław residents living in four-person households and 24% lower in two-person households than among respondents living in households with five or more people. Respondents without a steady source of income were 45% more likely to have a normal body weight compared to people with a steady source of income. The likelihood of normal body weight among Wrocław residents with a disposable net income of above 156 USD was 64%, 53–104 USD—48%, up to 52 USD—47%, and 105–145 USD—42% lower than in the respondents who did not have it. Respondents’ body weight was also significantly correlated with having money savings, as respondents with savings were 34% more likely to be of normal body weight than those with no savings (Table 4).

## 4. Discussion

The study results show that about 60% of the working-age respondents from Wrocław were of normal body weight. This corresponds to WHO criteria [1], according to which normal body weight is observed in 61.1% of the world population aged 18 years and over. The most recent study on the Polish population indicates that only about 40% of people had normal body weight [2]. However, it covers the Polish population as a whole, while, at present, the differences in the prevalence of overweight are increasingly more recognized in urban and rural populations. Moreover, the percentage of Poles with normal body weight in earlier studies also ranged from 45 to 60 percent [34]. However, it should be noted that apart from the place of residence, one of the potential reasons for the high percentage of people of normal body weight among the examined Wrocław residents could be related to the applied research methodology.

Among the Wrocław residents of working age, women were found to be of normal body weight significantly more often than men. The results of earlier studies also confirmed this observation [2,23,24,35,36]. Interestingly, men are usually characterized by higher physical activity levels than women [15,37]. However, some authors indicate that gender is a factor that significantly differentiates between the eating habits of adults and that men tend to follow a more unhealthy diet in comparison with women [38,39,40]. For example, Sulig et al. [41] revealed that men consumed meat and cold cuts, fast food, fried dishes, snacks, alcohol, and sugar-sweetened products and drinks more often, while women ate more fruit, milk, and dairy products. The key significance of diet for body weight and, consequently, the BMI in men was demonstrated by Lopez-Olmedo et al. [42]. Furthermore, according to Wronka et al. [43], women, more often than men, are not satisfied with their own body weight and take action to reduce it.

In the present study, normal body weight was found most often in younger respondents (18–34 years of age). Additionally, nationwide studies in Poland show that the prevalence of overweight increases with age [2]. A similar observation was made with regard to other populations by Hong et al. [20], Kolahi et al. [21], Rengma et al. [24], and Arredondo et al. [44]. Camhi et al. [45] reported healthier dietary habits among young people compared with the elderly, while significant links between the BMI and diet were demonstrated by Guo et al. [46] and Tande et al. [47]. The results of previous studies also indicate that the level of physical activity decreases with age [48]. This applies both to leisure-time physical activity as well as activities performed at work and while commuting [49]. Keith et al. [50] also indicated that social approval for overweight and obesity increases with age among members of a given peer group.

When considering the family situation of Wrocław inhabitants, it was noticed that the likelihood of normal body weight was higher among single individuals than among married people. Moreover, normal body weight was reported more often in individuals from households with two and four persons than from households with five and more persons. Hong et al. [20] also demonstrated a higher incidence of the normal BMI range in women aged 15–49 years living alone. In contrast, Micklesfield et al. [19] observed lower BMI values in men and higher BMI values in single women compared to those in relationships. Moreover, Kolahi et al. [21] demonstrated positive correlations between the prevalence of normal body weight and the number of people in the household. Therefore, this issue has not been fully explained yet, although studies have clearly indicated a higher level of physical activity among single people. This pertains primarily to leisure-time physical activity, which currently dominates the structure of physical activity of societies in developed countries [15,48].

The Wrocław residents’ body weight, assessed according to WHO criteria, was also associated with their socioeconomic status. Considering the respondents’ occupations, it was found that the lowest percentage of individuals of normal body weight was among the unemployed and the highest was among the manual workers. A higher frequency of normal body weight among the employed than among the unemployed was also reported by Micklesfield et al. [19] and Kolahi et al. [21]. The main reasons for this were probably the higher levels of physical activity of employed people compared to the unemployed, especially at work and while commuting [48]. Lack of income or earning lower income favored normal body weight, while the lack of savings was associated with higher BMI values. It should be stressed, however, that in other studies, the relationship between BMI values and financial situation was not clear. Micklesfield et al. [19], in their study of Johannesburg residents, noted higher BMI values in people with a better financial status. Similarly, Hong et al. [20], in their study of a Myanmar population, observed that higher income levels were often associated with the prevalence of overweight and obesity. Similar correlations between body weight and socioeconomic status were found by Kolahi et al. [21], Rengma et al. [24], Akinyemiju et al. [28], and Zujko et al. [51]. Negative correlations between body weight and socioeconomic status were reported by Pigeyre et al. [25]. Christine et al. [26], Araujo et al. [29], Wagner et al. [27], and Kriaucioniene et al. [23] noted that mean BMI values increased in men and decreased in women along with higher education and wealth levels. Interestingly, albeit not completely verifiable, Rengma et al. [24] claimed that the positive or negative correlations between body weight and socioeconomic status depend on the wealth level of the country of residence. Generally, they asserted that people with a higher socioeconomic status in developed countries, as well as people with a lower socioeconomic status in underdeveloped countries, tend to be of normal body weight more often. These differences result mainly from the greater availability of food and the more sedentary lifestyle of working-age people from rich countries than from poor ones. Equally ambiguous research results have been found in the case of links between physical activity and the socioeconomic status of adults. In some studies, people with a higher socioeconomic status were characterized by greater higher physical activity [51,52], while in others, the opposite appears to be the case [15]. Moreover, Zujko et al. [51] demonstrated that with rising socioeconomic status, the percentage of people of both sexes following a higher antioxidant intake diet increases. This may point to more rational nutrition of economically better-off individuals and to the necessity to reduce their body overweight. 

### Strengths and Limitations

The present article has its strengths and weaknesses. Its main strength is the consideration of socioeconomic status in terms of a wide range of variables. For the first time in population studies, factors such as having a steady source of income, disposable net income or savings, and debt have been taken into account as potential BMI determinants. There have also been very few studies covering such a broad age range and representative urban populations, especially from developed countries. The main weaknesses of the article are the cross-sectional nature of the research and its narrow spatial scope. Future studies should be of a continuous character and should cover the general populations from Poland and other Central European countries.

## 5. Conclusions

According to WHO criteria, three-fifths of the studied Wrocław residents, aged 18–64 years, were of normal body weight, while the remaining two-fifths were categorized as overweight. Occupational status, marital status, number of people in the household, having a steady source of income, disposable net income, and savings were significant determinants of body mass index. The objective of cross-sectional studies is to propose recommendations for public health action strategies. Therefore, it is necessary to recommend programs aimed at rational nutrition and physical activity improvement, addressed primarily to respondents from groups with the highest risk of overweight, to decision-makers. These programs should take into account the characteristics of the respondents’ occupations, such as physical effort, stress, and working conditions, or their coworkers’ impact in terms of lifestyle models and behavior patterns.

## Figures and Tables

**Table 1:** Demographic and socioeconomic characteristics of the Wrocław residents under study (*n* = 4315).

Variables	Category	Frequency	Percent	Chi-Square	*p*-Value
Sex	Woman	2206	51.12	2.18	0.140
	Man	2109	48.88		
Age	18–24 years	576	13.35	207.75	<0.001
	25–34 years	1119	25.93		
	35–44 years	867	20.09		
	45–54 years	752	17.43		
	55–64 years	1001	23.20		
Education	Primary	1680	38.93	138.02	<0.001
	Secondary	1553	35.99		
	Higher	1082	25.08		
Job	Manual worker	1142	26.47	538.28	<0.001
	White-collar worker	1315	30.48		
	Self-employed	605	14.02		
	Student	582	13.49		
	Unemployed	671	15.55		
Marital status	Single	1738	40.28	163.13	<0.001
	Married	2577	59.72		
Persons per household	1 person	546	12.65	578.54	<0.001
	2 persons	970	22.48		
	3 persons	1231	28.53		
	4 persons	1130	26.19		
	≥5 persons	438	10.15		
Steady income	No	812	18.82	1678.21	<0.001
	Yes	3503	81.18		
Per capita income	≤130 USD	295	6.84	558.32	<0.001
	131–260 USD	874	20.25		
	261–390 USD	1087	25.19		
	391–520 USD	866	20.07		
	>520 USD	1193	27.65		
Disposable (net) income	No income	381	8.83	513.09	<0.001
	≤52 USD	794	18.40		
	53–104 USD	1141	26.44		
	105–156 USD	786	18.22		
	>156 USD	1213	28.11		
Savings	No	2261	52.40	9.93	0.002
	Yes	2054	47.60		
Indebtedness	No	2268	52.56	11.32	0.001
	Yes	2047	47.44		

**Table 2:** Wrocław residents’ body mass following WHO criteria (*n* = 4315).

Variable	Category	Frequency	Percent	Chi-Square	*p*-Value
Body mass	Normal weight	2590	60.02	173.40	<0.001
	Overweight	1725	39.98		

**Table 3:** Body mass and selected demographic and socioeconomic characteristics of Wrocław residents (*n* = 4315).

Variable	Category	Body Mass				Chi-Square	*p*-Value	Cramer’s V
		Normal Weight		Overweight				
		Frequency	Percent	Frequency	Percent			
Sex	Woman	1575	71.40	631	28.60	243.30	<0.001	0.24
	Man	1015	48.13	1094	51.87			
Age	18–24 years	470	81.60	106	18.40	390.20	<0.001	0.30
	25–34 years	831	74.26	288	25.74			
	35–44 years	497	57.32	370	42.68			
	45–54 years	294	39.10	458	60.90			
	55–64 years	498	49.75	503	50.25			
Education	Primary	982	58.45	698	41.55	40.86	<0.001	0.10
	Secondary	1024	65.94	529	34.06			
	Higher	584	53.97	498	46.03			
Job	Manual worker	692	60.60	450	39.40	210.73	<0.001	0.22
	White-collar worker	711	54.07	604	45.93			
	Self-employed	292	48.26	313	51.74			
	Student	497	85.40	85	14.60			
	Unemployed	398	59.31	273	40.69			
Marital status	Single	1265	72.78	473	27.22	197.52	<0.001	0.21
	Married	1325	51.42	1252	48.58			
Persons per household	1 person	327	59.89	219	40.11	33.49	<0.001	0.09
	2 persons	519	53.51	451	46.49			
	3 persons	728	59.14	503	40.86			
	4 persons	741	65.58	389	34.42			
	≥5 persons	275	62.79	163	37.21			
Steady income	No	602	74.14	210	25.86	83.05	<0.001	0.14
	Yes	1988	56.75	1515	43.25			
Per capita income	≤130 USD	211	71.53	84	28.47	75.12	<0.001	0.13
	131–260 USD	575	65.79	299	34.21			
	261–390 USD	656	60.35	431	39.65			
	391–520 USD	544	62.82	322	37.18			
	>520 USD	604	50.63	589	49.37			
Disposable net income	No income	275	72.18	106	27.82	106.55	<0.001	0.16
	≤52 USD	515	64.86	279	35.14			
	53–104 USD	715	62.66	426	37.34			
	105–156 USD	498	63.36	288	36.64			
	>156 USD	587	48.39	626	51.61			
Savings	No	1388	61.39	873	38.61	3.69	0.055	0.03
	Yes	1202	58.52	852	41.48			
Indebtedness	No	1423	62.74	845	37.26	14.73	<0.001	0.06
	Yes	1167	57.01	880	42.99			

**Table 4:** Logistic model of body mass following WHO criteria with regard to selected demographic and socioeconomic characteristics of Wrocław residents (*n* = 4315).

Variable	Category	B	Std. Error	Wald	*p*-Value	Exp (B)	95% Confidence Interval for Exp(B)	
							Lower Bound	Upper Bound
Intercept		−0.17	0.18	0.88	0.347			
Sex	Woman	1.31	0.08	284.47	<0.001	3.70	3.18	4.30
	Man	0 ^a^						
Age	18–24 years	1.20	0.15	60.46	<0.001	3.32	2.45	4.50
	25–34 years	0.89	0.11	62.27	<0.001	2.43	1.95	3.04
	35–44 years	0.30	0.11	8.01	0.005	1.35	1.10	1.66
	45–54 years	−0.55	0.11	26.38	<0.001	0.58	0.47	0.71
	55–64 years	0 ^a^						
Job	Manual worker	0.91	0.14	43.17	<0.001	2.48	1.89	3.25
	White collar worker	0.48	0.13	12.96	<0.001	1.62	1.25	2.10
	Self-employed	0.58	0.16	13.37	<0.001	1.78	1.31	2.43
	Student	0.83	0.17	23.60	<0.001	2.29	1.64	3.20
	Unemployed	0 ^a^						
Marital status	Single	0.25	0.09	7.16	0.007	1.29	1.07	1.55
	Married	0 ^a^						
Persons per household	1 person	0.04	0.16	0.06	0.811	1.04	0.76	1.42
	2 persons	−0.28	0.13	4.27	0.039	0.76	0.58	0.99
	3 persons	0.02	0.13	0.03	0.863	1.02	0.79	1.32
	4 persons	0.29	0.13	4.96	0.026	1.34	1.04	1.74
	≥5 persons	0 ^a^						
Steady income	Yes	−0.60	0.13	21.37	<0.001	0.55	0.43	0.71
	No	0 ^a^						
Net income	≤52 USD	−0.64	0.16	16.81	<0.001	0.53	0.39	0.72
	53–104 USD	−0.66	0.15	19.49	<0.001	0.52	0.38	0.69
	105–156 USD	−0.55	0.16	11.49	0.001	0.58	0.42	0.79
	>156 USD	−1.03	0.16	43.76	<0.001	0.36	0.26	0.48
	No income	0 ^a^						
Savings	Yes	0.29	0.08	13.82	<0.001	1.34	1.15	1.57
	No	0 ^a^						

^a^ This parameter is set to zero because it is redundant. Likelihood ratio tests (chi-square = 884.01, df = 20, Sig. < 0.001); R^2^_Nagelkerke_ = 0.25).
